# Cognitive Outcomes After Transcranial Magnetic Stimulation for the Treatment of Late-Life Depression: Résultats cognitifs après la stimulation magnétique transcrânienne pour le traitement de la dépression chez les personnes âgées

**DOI:** 10.1177/07067437251315515

**Published:** 2025-01-29

**Authors:** Katharina Göke, Shawn M. McClintock, Linda Mah, Tarek K. Rajji, Hyewon H. Lee, Sean M. Nestor, Jonathan Downar, Yoshihiro Noda, Zafiris J. Daskalakis, Benoit H. Mulsant, Daniel M. Blumberger

**Affiliations:** 1 Temerty Centre for Therapeutic Brain Intervention and Campbell Family Research Institute, 7978 Centre for Addiction and Mental Health, Toronto, ON, Canada; 2 Institute of Medical Science, 7938 University of Toronto, Toronto, ON, Canada; 3 Division of Psychology, Department of Psychiatry, University of Texas Southwestern Medical Center, Dallas, TX, USA; 4 Department of Psychiatry, Temerty 12366 Faculty of Medicine, University of Toronto, Toronto, ON, Canada; 5 Rotman Research Institute, Baycrest Health Sciences, Toronto, ON, Canada; 6 Toronto Dementia Research Alliance, 7938 University of Toronto, Toronto, ON, Canada; 7 Harquail Centre for Neuromodulation, 282299 Sunnybrook Health Sciences Centre, Toronto, ON, Canada; 8 Department of Neuropsychiatry, 38084 Faculty of Medicine, Keio University School of Medicine, Tokyo, Japan; 9 Department of Psychiatry, 8784 University of California, San Diego Health, CA, USA

**Keywords:** late-life depression, repetitive transcranial magnetic stimulation, cognition, executive function, reliable change index

## Abstract

**Background:**

Late-life depression (LLD) is often accompanied by cognitive impairment, which may persist despite antidepressant treatment. Repetitive transcranial magnetic stimulation (rTMS) is an efficacious treatment for depression, with potential benefits on cognitive functioning. However, research on cognitive effects is inconclusive, relatively sparse in LLD, and predominantly focused on group-level cognitive changes. This study aimed to explore individual-level cognitive changes following rTMS treatment in patients with LLD.

**Method:**

Data were analyzed from 153 patients with LLD from the FOUR-D study (ClinicalTrials.gov identifier: NCT02998580) who received bilateral standard rTMS or theta burst stimulation (TBS) targeting the dorsolateral prefrontal cortex (DLPFC). Cognitive function was assessed pre- and post-treatment using measures of executive function, information processing speed, and learning and memory. Reliable change indices, adjusted for practice effects and test-retest reliability, were employed to evaluate individual-level cognitive changes. Chi-square tests examined if proportions of cognitive improvers differed from expected proportions.

**Results:**

Cognitive performance from baseline to end of treatment remained stable for most patients. Reliably improved performance was observed in 0.0% to 20.0% of participants across cognitive measures, while worsened performance was observed in 0.0% to 2.7%. A small but significant proportion (20.0%) of participants showed improvement in verbal learning.

**Conclusions:**

Bilateral standard rTMS or TBS of the DLPFC in LLD yielded no substantial cognitive enhancing effects, although a small proportion showed improved verbal learning after treatment. Importantly, both interventions were cognitively safe with relatively stable performance across time. Future research is needed to explore approaches to enhance the cognitive benefits of standard rTMS and TBS in patients with LLD.

## Résumé

**Contexte:**

la dépression chez les personnes âgées est souvent accompagnée de troubles cognitifs, qui peuvent persister malgré un traitement aux antidépresseurs. La stimulation magnétique transcrânienne répétitive (SMTr) est un traitement efficace de la dépression, qui pourrait avoir des effets bénéfiques sur le fonctionnement cognitif. Cependant, les recherches sur les effets cognitifs ne sont pas concluantes, elles sont relativement rares dans le cas de la dépression chez les personnes âgées et se concentrent principalement sur les changements cognitifs au niveau du groupe. Cette étude visait à explorer les changements cognitifs survenus au niveau individuel à la suite d'un traitement par SMTr chez des personnes âgées souffrant de dépression.

**Méthode:**

les données issues de l'étude FOUR-D (ClinicalTrials.gov identifier : NCT02998580) et portant sur 153 patients âgés atteints de dépression qui ont reçu une SMTr bilatérale standard ou une stimulation thêta-burst (STB) ciblant le cortex préfrontal dorsolatéral (CPFdl) ont été analysées. La fonction cognitive a été évaluée avant et après le traitement à l'aide de mesures de la fonction exécutive, de la vitesse de traitement de l'information, de l'apprentissage et de la mémoire. Des indices de changement fiables, ajustés pour tenir compte des effets de la pratique et de la fiabilité test-retest, ont été utilisés pour évaluer les changements cognitifs individuels. Des tests du chi-carré ont permis de vérifier si les proportions de personnes ayant amélioré leurs capacités cognitives différaient des proportions attendues.

**Résultats:**

les performances cognitives au début de l'étude et à la fin du traitement sont restées stables chez la plupart des patients. Une amélioration fiable des performances a été observée chez 0,0 % à 20,0 % des participants sur l'ensemble des mesures cognitives, tandis qu'une détérioration des performances a été observée chez 0,0 % à 2,7 % d'entre eux. Une proportion faible, mais significative (20,0 %) de participants a affiché une amélioration de l'apprentissage verbal.

**Conclusions:**

la SMTr bilatérale standard ou la STB ciblant le CPFdl chez les personnes âges souffrant de dépression n'a pas produit d'effets cognitifs substantiels, bien qu'une petite proportion d'entre elles aient présenté une amélioration de l'apprentissage verbal après le traitement. Il est important de mentionner que les deux interventions étaient sûres sur le plan cognitif et que les performances étaient relativement stables dans le temps. Des recherches futures sont nécessaires pour explorer d'autres approches permettant d'améliorer les avantages cognitifs de la SMTr standard et de la STB chez les patients âgés souffrant de dépression.

## Introduction

Late-life depression (LLD) is often accompanied by cognitive impairment, particularly in the domains of executive functioning, information processing speed, and learning and memory.^1^ These impairments significantly reduce quality of life and increase the risk of neurodegenerative disorders such as Alzheimer's disease and related dementias.^2^ Antidepressant medications are widely used in treating LLD, yet tend to be only partially beneficial for many patients who still have significant depressive symptoms.^3^ Importantly, cognitive impairments often persist even when there is improvement in depressive symptoms and severity.^4,5^ While a recent meta-analysis suggested potential improvements in learning and memory, mediated by alleviation of depressive symptoms,^6^ the overall effects of antidepressants on cognitive function in LLD remain controversial.^7^ This highlights the need for interventions that can effectively address both depressive and cognitive symptoms in LLD.

Repetitive transcranial magnetic stimulation (rTMS) of the dorsolateral prefrontal cortex (DLPFC) is a well-established treatment for patients with treatment-resistant depression (TRD). The standard approach involves high-frequency rTMS (10 Hz) over the left DLPFC, and/or low-frequency rTMS (1 Hz) over the right DLPFC.^8^ Another form of rTMS, theta burst stimulation (TBS), replicates natural brain oscillatory activity through patterned pulse trains, and is delivered as intermittent (iTBS) or continuous (cTBS) bursts over the left or right DLPFC, respectively.^8^ Evidence indicates that TBS is comparably effective to standard rTMS for depression.^9^ While high-frequency rTMS and iTBS are thought to exert excitatory effects on neuronal activity, low-frequency rTMS and cTBS are believed to be inhibitory, although these effects show substantial inter-individual variability.^10^ In contrast to electroconvulsive therapy (ECT), rTMS is recognized for its cognitive safety^11^ and potential to improve certain cognitive functions in TRD.^12^ The DLPFC is a key node within the cognitive control network,^13^ which is thought to subserve executive functions, such as response inhibition, set-shifting, and planning. rTMS uses focused magnetic field pulses to modify neural activity at the stimulation site and can induce structural and functional connectivity changes in distributed brain networks,^14^ including the cognitive control network.^15^ By targeting the DLPFC, rTMS may have the potential to improve executive function in patients with depression.

Cognitive improvements following rTMS have been observed in younger adults with depression across various domains including learning and memory,^16–18^ attention,^19^ executive function^19–24^ and psychomotor processing speed.^22,25,26^ However, findings have been inconsistent and large meta-analyses have not demonstrated robust cognitive effects,^27^ with only modest effects observed in specific cognitive tasks.^28^ Research on cognitive effects of rTMS in LLD is limited, despite potential differences within this age group. Some rTMS studies in LLD have reported improvements in attention and immediate memory,^29^ executive function,^30,31^ verbal fluency and visuospatial abilities,^32^ and global cognitive function.^33^ However, other studies found no significant changes in cognitive function after treatment.^34–36^

Studies investigating cognitive changes following rTMS have primarily relied on group-level analyses, which may mask changes at the individual (i.e., patient) level. Practice-adjusted reliable change indices (RCI_PE_) can be used to detect improved or worsened cognitive performance in individuals beyond measurement error and practice effects,^37^ which can address this limitation. In this context, we investigated individual-level cognitive changes associated with a course of bilateral standard rTMS or TBS to the DLPFC in patients with LLD using an RCI_PE_ methodology. We hypothesized that bilateral rTMS/TBS treatment would enhance cognitive function, particularly executive function. Additionally, we explored potential predictors of cognitive improvement and whether cognitive changes varied across different baseline cognitive subgroups.

## Methods

### Participants

Cognitive data was analyzed from the FOUR-D study, a randomized, non-inferiority trial comparing bilateral TBS to bilateral standard rTMS in patients with LLD (ClinicalTrials.gov identifier: NCT02998580).^9^ The study received approval from the CAMH Research Ethics Board and enrolled outpatients age 60 and older with Major Depressive Disorder. Detailed inclusion and exclusion criteria have been previously reported.^9^ In brief, participants were required to show non-response to at least one antidepressant trial at an adequate dosage and duration, or intolerance to at least two antidepressants, and have a Montgomery Åsberg Depression Rating Scale (MADRS)^38^ total score ≥ 18. Prior to their participation in the study, all participants provided written informed consent.

### Cognitive Assessment

Trained personnel conducted the following cognitive tests at baseline and post-treatment:

- The NIH Toolbox Flanker Inhibitory Control and Attention Test assessed executive function, specifically selective attention and inhibitory control.^39^ Participants were instructed to respond to a central stimulus while ignoring similar surrounding stimuli. Scores were computed based on accuracy and reaction times and then converted into age-adjusted standard scores (M = 100, SD = 15).
- The Delis-Kaplan Executive Function System (D-KEFS) Color-Word Interference test (CWI)^40^ was conducted including four conditions: The two baseline conditions Color Naming (Condition 1) and Word Reading (Condition 2) were used as measures of information processing speed. Executive functioning was evaluated using the Interference condition (Condition 3), measuring response inhibition, and the Inhibition/Switching condition (Condition 4), measuring both inhibition and cognitive flexibility. Raw scores (i.e., time taken to complete the task) for all four conditions were then converted into age-adjusted scaled scores (M = 10, SD = 3).
- The California Verbal Learning Test – second edition (CVLT-II)^41^ was used to assess verbal learning and memory. Participants were presented with a list of 16 semantically related words over 5 learning trials, with verbal learning scores derived from the total number of correctly recalled words over these trials. Subsequently, a short-delay free recall assessed the number of correctly recalled words after an interference list. This was followed by a long-delay free recall after a 20-min interval. Raw CVLT-II scores were converted into normative T- and z-scores adjusted for age and reported sex.

Additional cognitive measures included the Montreal Cognitive Assessment (MoCA version 2),^42^ which evaluated overall global cognitive function. The Test of Premorbid Functioning (TOPF),^43^ a word reading test, was used to estimate premorbid IQ at baseline.

### Clinical Assessment

Clinical assessments included the MADRS to assess depressive symptom severity, the Cumulative Illness Rating Scale-Geriatrics (CIRS-G)^44^ to evaluate burden of physical illness, and the Brief Symptom Inventory (BSI)–Anxiety Subscale (BSI-Anxiety).^45^ Additional clinical variables encompassed age at first onset of depression, duration of current depressive episode, details regarding current medications, and adequacy of current and previous antidepressant medications as quantified using the Antidepressant Treatment History Form (ATHF).^46^

### Procedures

Participants were randomized to receive either standard sequential bilateral rTMS starting with right-sided 1 Hz stimulation (10 min for 600 pulses), followed by left-sided 10 Hz stimulation (37.5 min for 3000 pulses, 4 s on, 26 s off) or sequential bilateral TBS starting with right-sided cTBS (40 s for 600 pulses in 50 Hz triplet bursts, repeated at 5 Hz) followed by left-sided iTBS (3 min 9 s for 600 pulses in 50 Hz triplet bursts, repeated at 5 Hz, 2 s on, 8 s off). All treatments were administered at 120% resting motor threshold to the DLPFC and delivered 5 days a week over 4 weeks, totaling 20 treatment sessions. If remission was not achieved after the first 20 sessions, 10 additional sessions over the following 2 weeks were offered.

### Statistical Analysis

This analysis included all participants with available cognitive data at both time points for at least one cognitive measure to allow for the calculation of change scores. Baseline demographic and clinical characteristics of the sample were summarized using means and standard deviations (SD) for continuous variables, and frequencies for categorical variables. To account for measurement error and practice effects inherent in repeated neuropsychological assessments, we used RCI_PE_^37^ because of its demonstrated usefulness in assessing cognitive change in older adults compared to alternative change score methods.^47^ RCI_PE_ were computed by subtracting the mean practice effect (derived from a normative sample of the respective test) from the individuals’ observed discrepancy score (time 2−time 1), divided by the standard error of the difference (SED):

$$RCI=\frac{\left(X_{2}-X_{1})-(M_{2}-M_{1}\right)}{SED}$$

Here, X_1_ and X_2_ represent the individual's observed baseline and end of treatment cognitive test scores, respectively, while M_1_ and M_2_ denote the normative group mean baseline and follow-up scores. The SED was calculated according to the adjusted formula^48^:

$$SED=\sqrt{\left(SD_{1}\sqrt{1-\;r_{12}}\;\right){\;}^{2}+\left(SD_{2}\sqrt{1-\;r_{12}}\;\right){\;}^{2}}$$

Where SD_1_ and SD_2_ represent the standard deviations at time 1 and time 2, and r_12_ is the test-retest reliability, both obtained from normative test-retest data. Normative data was extracted from three psychometric papers providing normative scores and test-retest reliabilities for the NIH Flanker Task,^49^ DKEFS CWI,^40^ and CVLT-II.^50^ Age-adjusted scaled and standard scores were used for the NIH Flanker Task and DKEFS-CWI, while only raw scores were available for the test-retest data of the CVLT-II. To identify if a patient's cognitive score has significantly changed beyond measurement error and practice effects, a 90% confidence interval for reliable change was employed, consistent with prevailing RCI literature.^51,52^ RCI z-scores had to be 1.645 or higher to be considered a “reliable improvement,” values of −1.645 or lower were considered a “reliable decline,” and all values within the 90% confidence interval were considered “no change.” Assuming that RCI z-scores are normally distributed, then it is expected that 5% of participants show “decline” (i.e., smaller than expected improvement), 90% would show “no change” (i.e., expected levels of improvement), and 5% would show “improvement” (i.e., larger than expected improvement). One-sample chi-square tests were used to examine if observed frequencies of participants classified as “reliable improvement” significantly exceed the expected number of participants with “reliable improvement” (i.e., 5%).

For cognitive tests with a significant proportion of participants showing a “reliable improvement,” further exploratory analyses examined demographic and clinical predictors of cognitive improvement using binary logistic regression with backward elimination method based on Akaike's Information Criterion. Baseline performance of the respective test was entered as a covariate, and potential predictive variables included sex, age, years of education, estimated premorbid IQ, baseline MADRS, baseline BSI-A, ATHF score, duration of current depressive episode, benzodiazepine use (y/n), treatment allocation and number of rTMS treatments delivered. To assess potential associations with effects of rTMS on depressive symptoms, change in MADRS total score (post-treatment MADRS score−baseline MADRS score), response (reduction ≥ 50% from baseline in MADRS score), and remission (MADRS score ≤ 10 at end of treatment) were included as additional predictors. Additionally, Spearman's rank correlation between the RCI z-score and MADRS change score was calculated.

To investigate how cognitive changes vary depending on an individual's initial cognitive profile, we examined reliable changes across three distinct baseline cognitive subgroups. These subgroups were identified in a previous study using a k-means clustering approach based on baseline cognitive test scores from the current sample.^53^ Specifically, the cluster analysis revealed 3 distinct cognitive subgroups labeled as “Cognitively Intact” (*n *= 84), “Cognitively Diminished” (*n *= 25), and “Impaired Memory” (*n *= 39). We examined whether RCI z-scores differed significantly across these three cognitive subgroups using ANOVA. We also used chi-square tests to examine whether proportions of participants showing reliable improvement, no change, or decline differed across the cognitive subgroups. A false discovery rate (FDR) correction was applied to account for multiple tests across cognitive measures and *p*-values < 0.05 were considered statistically significant. All statistical analyses were conducted using R (version 4.1.2).

## Results

Out of 172 randomized participants, 8 participants did not complete treatment, and another 11 participants did not undergo post-treatment cognitive assessment, resulting in the inclusion of 153 participants in this analysis who were assessed at both time points on at least one cognitive measure (mean [SD] age, 66.7 [6.0] years; 56% female, Table 1). There were no significant differences in demographic or clinical variables between participants lost to follow-up and those who underwent both assessments. However, participants who did not complete treatment or undergo post-treatment cognitive assessment showed significantly worse performance at baseline on the CVLT-II Total Trials 1–5 (*t*(19.81) = 3.68, *p *= 0.001), the CVLT-II Short-Delay Free Recall (*t*(19.05) = 2.73, *p *= 0.013), and the CVLT-II Long-Delay Free Recall (*t*(18.44) = 3.12, *p *= 0.006).

**Table 1.** Baseline Demographic and Clinical Characteristics of the Study Sample.

		LLD patients (*n *= 153)
Treatment allocation, n (%)	*TBS*	71 (46%)
	*rTMS*	82 (54%)
Age, mean (SD)		66.7 (6.0)
Self-reported sex, n (%)	*Male*	68 (44%)
	*Female*	85 (56%)
Years of education, mean (SD)		15.39 (2.62)
MADRS total score, mean (SD)		25.8 (4.7)
BSI-A score, mean (SD)		10.3 (5.4)
Age at onset, mean (SD)		31 (19)
Medication trials, mean (SD)		2.2 (1.45)
Episode length (months), mean (SD)		59 (90)
CIRS-G total score, mean (SD)		5.6 (3.2)
MoCA total score, mean (SD)		25.2 (3.1)
TOPF age-corrected standard score, mean (SD)		112 (10)
Receiving pharmacotherapy during treatment, n (%)		
No antidepressant medication		23 (15%)
Hypnotic Z drugs		31 (20%)
Benzodiazepine		65 (42%)
Antidepressant alone		59 (39%)
Antidepressant combination		31 (20%)
Augmentation		33 (22%)
Antipsychotic alone		2 (1.3%)
Lithium augmentation		8 (5.2%)
Stimulant		13 (8.5%)

Abbreviations: BSI-A, Brief Symptom Inventory–Anxiety; CIRS-G, Cumulative Illness Rating Scale-Geriatric; LLD, late-life depression; MOCA, Montreal Cognitive Assessment; Montgomery-Åsberg Depression Rating Scale; TOPF, Test of Premorbid Function.

### Practice-Adjusted Reliable Change Index

**Figure 1.**
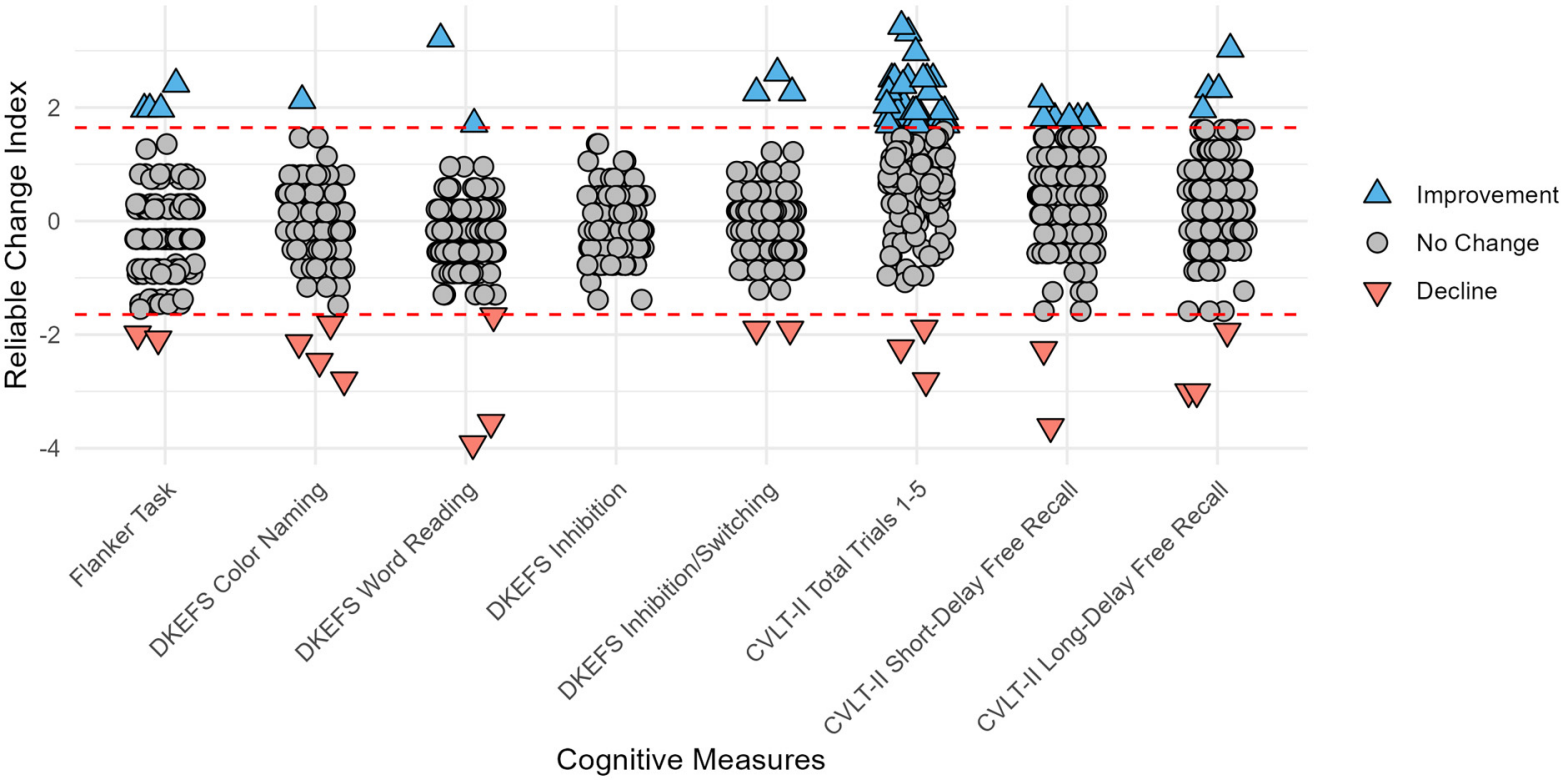
Distribution of RCI_PE_ z-scores. Dashed lines indicate 90% confidence interval. Abbreviations: CVLT-II, California Verbal Learning Test – second edition; DKEFS, Delis-Kaplan Executive Function System.

Most participants showed no reliable change in performance on cognitive measures (Table 2). The percentage of participants showing a reliable improvement ranged across cognitive measures from 0.0% (on the DKEFS-CWI Inhibition Task) to 20.0% (on the CVLT-II Total Trials 1–5). Overall, 25.5% (39/153) of participants showed a reliable improvement in at least one cognitive measure. Conversely, the percentage of participants showing a reliable worsened performance ranged from 0.0% (on the DKEFS-CWI Inhibition Task) to 2.7% (on the DKEFS-CWI Color Task) (Table 2). Distributions of RCI z-scores for each cognitive measure are depicted in Figure 1. Notably, for most cognitive measures, the percentage of participants exhibiting a reliable increase or decrease in performance fell within the expected 5% range based on the distribution of RCIs. This was exceeded only by the percentage of participants who improved in verbal learning on the CVLT-II Total Trials 1–5, where 30 out of 150 participants (20.0%) had reliably improved performance. This difference between the observed percentage with the expected percentage was significant (χ2(2) = 48.4, *p *< 0.001).

**Table 2.** Cognitive Scores at Baseline and End of Treatment and Frequency (Percentage) of Participants Showing Reliable Cognitive Changes Using the RCI_PE_ Method With a 90% Confidence Interval.

	Baseline		End of treatment		Improved	No change	Declined
	*N*	Mean (SD)	*N*	Mean (SD)	*n* (%)	*n* (%)	*n* (%)
Flanker Task age-corrected standard score	153	88 (11)	148	89 (12)	4 (2.7%)	142 (95.9%)	2 (1.4%)
DKEFS							
Color Naming Scaled Score	153	9.2 (3.3)	150	9.2 (3.3)	1 (0.7%)	145 (96.7%)	4 (2.7%)
Word Reading Scaled Score	153	10.44 (2.82)	150	10.25 (2.86)	2 (1.3%)	145 (96.7%)	3 (2.0%)
Inhibition Scaled Score	151	10.40 (2.85)	150	10.67 (2.83)	0 (0.0%)	148 (100%)	0 (0.0%)
Inhibition/Switching Scaled Score	150	11.06 (2.62)	147	11.41 (2.71)	3 (2.1%)	139 (96.5%)	2 (1.4%)
CVLT-II							
Total Trials 1–5 T-Score	151	49 (10)	150	57 (13)	30 (20.0%)	117 (78%)	3 (2.0%)
Short-Delay Free Recall Z Score	151	−0.06 (1.11)	150	0.42 (1.24)	7 (4.7%)	141 (94%)	2 (1.3%)
Long-Delay Free Recall Z Score	151	−0.16 (1.08)	149	0.32 (1.19)	4 (2.7%)	142 (95.3%)	3 (2.0%)

Abbreviations: CVLT-II, California Verbal Learning Test – second edition; DKEFS, Delis-Kaplan Executive Function System; LLD, late-life depression.

### Exploratory Prediction Analysis

A binary logistic regression analysis using backward elimination was conducted to determine significant predictors of a reliable improvement on the CVLT-II Total Trials 1–5. The final model retained three significant predictors. A lower baseline performance on this measure (*p *= 0.01, OR = 0.94, CI 0.90–0.98), a higher premorbid IQ (*p *= 0.01, OR = 1.07, CI 1.02–1.13), and being a responder to rTMS/TBS treatment (*p *= 0.04, OR = 2.48, CI 1.05–6.00) were associated with higher odds of showing a reliable improvement on the CVLT-II Total Trials 1–5. No other variable significantly predicted reliable improvement CVLT-II Total Trials 1–5, including treatment allocation (i.e., standard rTMS vs TBS). There was a weak but significant correlation between the RCI z-scores of the CVLT-II Total Trials 1–5 and the MADRS change score (ρ = −0.23, *p* = 0.005).

### Baseline Cognitive Subgroups

After FDR-correction, no significant differences in RCI z-scores were found across the three baseline cognitive subgroups in any cognitive measure, nor in percentages of participants showing reliable improvement, no change, or worsened performance (Table 3).

**Table 3.** Mean RCI z-Scores and Proportions of Participants Showing Reliable Improvement, No Change, or Decline for Three Baseline Cognitive Subgroups.

	Cluster 1 – Cognitively Intact (*n *= 84)	Cluster 2 – Cognitively Diminished (*n *= 25)	Cluster 3 – Impaired Memory (*n *= 39)	*p*-value (uncorrected)	*p*-value (FDR-corrected)
Flanker Task, mean (SD)	−0.15 (0.73)	−0.07 (0.70)	−0.42 (0.81)	0.111	0.444
Declined	0 (0.00%)	0 (0.00%)	2 (5.13%)	0.242	0.424
No change	79 (96.3%)	24 (100%)	36 (92.3%)		
Improved	3 (3.66%)	0 (0.00%)	1 (2.56%)		
DKEFS color naming, mean (SD)	−0.25 (0.58)	0.18 (0.76)	−0.06 (0.72)	0.012	0.096
Declined	2 (2.44%)	0 (0.00%)	2 (5.13%)	0.231	0.424
No change	80 (97.6%)	24 (96.0%)	37 (94.9%)		
Improved	0 (0.00%)	1 (4.00%)	0 (0.00%)		
DKEFS word reading, mean (SD)	−0.22 (0.42)	−0.12 (1.23)	−0.31 (0.78)	0.584	0.804
Declined	1 (1.22%)	1 (4.00%)	1 (2.56%)	0.043	0.301
No change	81 (98.8%)	22 (88.0%)	38 (97.4%)		
Improved	0 (0.00%)	2 (8.00%)	0 (0.00%)		
DKEFS inhibition, mean (SD)	−0.06 (0.44)	0.03 (0.72)	−0.09 (0.43)	0.603	0.804
Declined	0 (0.00%)	0 (0.00%)	0 (0.00%)	.	.
No change	82 (100%)	25 (100%)	39 (100%)		
Improved	0 (0.00%)	0 (0.00%)	0 (0.00%)		
DKEFS inhibition/Switching, mean (SD)	0.01 (0.51)	−0.14 (0.53)	−0.06 (0.82)	0.564	0.804
Declined	0 (0.00%)	0 (0.00%)	2 (5.26%)	0.097	0.340
No change	80 (98.8%)	24 (100%)	34 (89.5%)		
Improved	1 (1.23%)	0 (0.00%)	2 (5.26%)		
CVLT-II Total Trials 1–5, mean (SD)	0.91 (0.96)	0.75 (0.96)	0.74 (0.95)	0.593	0.804
Declined	2 (2.38%)	0 (0.00%)	0 (0.00%)	0.940	0.940
No change	64 (76.2%)	21 (84.0%)	31 (79.5%)		
Improved	18 (21.4%)	4 (16.0%)	8 (20.5%)		
CVLT-II Short-Delay Free Recall, mean (SD)	0.27 (0.82)	0.36 (1.02)	0.32 (0.67)	0.867	0.872
Declined	1 (1.19%)	1 (4.00%)	0 (0.00%)	0.616	0.719
No change	78 (92.9%)	23 (92.0%)	38 (97.4%)		
Improved	5 (5.95%)	1 (4.00%)	1 (2.56%)		
CVLT-II Long-Delay Free Recall, mean (SD)	0.34 (0.79)	0.44 (0.83)	0.38 (0.93)	0.872	0.872
Declined	2 (2.38%)	0 (0.00%)	0 (0.00%)	0.467	0.654
No change	81 (96.4%)	23 (95.8%)	37 (94.9%)		
Improved	1 (1.19%)	1 (4.17%)	2 (5.13%)		

Abbreviations: CVLT-II, California Verbal Learning Test – second edition; DKEFS, Delis-Kaplan Executive Function System; LLD, late-life depression.

## Discussion

Despite clear evidence for the therapeutic effects of standard rTMS and TBS in LLD, their effects on cognition remain unclear. In this study, we investigated the cognitive outcomes from the FOUR-D trial, a recent non-inferiority trial that compared bilateral standard rTMS with bilateral TBS to the DLPFC in patients with LLD. RCI_PE_ were used to effectively control for measurement error and practice effects of repeated neuropsychological testing. Overall, our findings indicated that bilateral standard rTMS or TBS to the DLPFC had limited effects on cognition across the domains of executive function and information processing speed. A small but statistically significant proportion of participants (i.e., 20%) experienced reliable improvements in verbal learning, which was associated with a higher estimated premorbid IQ and being a responder to rTMS treatment.

The literature on adults across the lifespan reveal mixed previous results regarding cognitive improvements after rTMS. For example, a transdiagnostic meta-analysis including depression reported positive rTMS effects on working memory, but found no effects on other cognitive domains.^54^ Another review of 31 RCTs reported positive effects on executive function and attention in some adults with depression.^55^ In the context of TRD, a review of 22 studies found a trend supporting that rTMS improves cognitive performance, although some studies reported negative findings.^12^ In line with our results, a meta-analysis of 30 RCTs of various neuropsychiatric disorders concluded that, overall, rTMS has no robust cognitive enhancing effects.^27^ However, a subsequent meta-analysis from the same authors focused on depression and revealed task-dependent improvements in aspects of executive function (i.e., observed on the TMT A and B), suggesting that measures used in different studies may not always capture the specific cognitive effects of rTMS.^28^

Contrary to our hypothesis, we observed no significant improvements in executive function among patients with LLD. Some research has suggested that executive function benefits of rTMS may be more pronounced in patients (and particularly older adults) with underlying executive dysfunction.^22,35,54^ While a previous meta-analysis found no significant age-related effects of rTMS on executive function in depression,^35^ a qualitative review of the literature suggested that, compared to younger adults, older adults with depression experience more notable improvements in executive function.^35^ In an open-label study with older adults with depression and co-existing executive dysfunction, bilateral iTBS led to significant improvements in executive function,^30^ though this finding was not replicated in the subsequent randomized sham-controlled trial.^34^ Similarly, other studies of rTMS, including deep TMS, found no improvements in executive function in LLD.^36,56,57^ Taken together, these findings suggest that DLPFC-rTMS may have no or weak beneficial impacts on executive function. This lack of a substantial effect may be due to the focus on treating depression rather than executive dysfunction, as well as the relatively short treatment duration and assessment periods used in these studies.

Although only a minority of participants exhibited reliable improvements in verbal learning, this aligns with several previous studies in older^29^ and younger adults^16,17,58^ with depression. Notably, improvement in verbal learning was predicted by a higher premorbid IQ which is indicative of greater cognitive reserve and may facilitate cognitive recovery or improvement after rTMS. Moreover, a reliable improvement in verbal learning was more likely in patients who responded to treatment (i.e., showed a reduction ≥ 50% from baseline in MADRS score). Previous research across various treatments, including pharmacological and psychological interventions, has linked improvements in learning and memory to reductions in depressive symptoms.^6^ This association has also been observed in prior rTMS studies,^17,36^ suggesting that improvements in verbal learning may be secondary to improvement in mood. However, the relationship between improvement in mood and cognition following rTMS remains inconsistent across cognitive domains. Some studies have linked cognitive improvements to reductions in depressive symptoms,^22,35^ while others found cognitive benefits independent of mood improvement,^28,31,59,60^ suggesting divergent mechanisms underlying depressive symptoms and cognitive function. Treatment allocation—whether participants received standard bilateral rTMS or bilateral TBS—did not significantly predict improvements in verbal learning. TBS is believed to induce more robust changes in synaptic plasticity compared to standard rTMS, as it more closely replicates the brain's natural firing patterns.^61^ In healthy adults, iTBS has demonstrated greater cognitive enhancement, particularly in working memory and executive function, when compared to high-frequency rTMS.^62^ However, both treatments showed similar effectiveness in improving depressive symptoms,^9^ which aligns with the lack of superior cognitive results from TBS in this study.

While our study observed no robust cognitive improvement after rTMS, we found no evidence of significantly worsened performance on any cognitive measure. Across all measures, the percentage of participants who experienced a reliable cognitive decline ranged from 0% to 2.7%, which is below the 5% expected to be due solely to measurement error. These findings reinforce the extensive literature demonstrating the cognitive safety of rTMS,^55^ supporting its use in treating LLD without the risk of cognitive decline. This makes rTMS preferable to other treatments (e.g., ECT, antipsychotics), which may have cognitive side effects.

It is important to note that rTMS primarily aimed to alleviate depressive symptoms. To address both depression and cognitive impairment in LLD, rTMS treatment may need to be designed accordingly. Similar to optimizing the site of stimulation to enhance therapeutic effects for depressive symptoms, identifying optimal targets within the DLPFC that modulate cognition (e.g., through neuroimaging) could be beneficial.^63,64^ Exploring alternative target sites beyond the DLPFC,^65^ such as the dorsomedial prefrontal cortex,^65,66^ or augmenting DLPFC stimulation with other targets,^67^ may also enhance rTMS cognitive effects. Recent research indicates that combining rTMS with transcranial direct current stimulation (tDCS), compared to rTMS alone, significantly enhances cognitive function and reduces clinical symptoms in younger adults with depression.^68^ Another promising strategy involves combining rTMS with cognitive training, which improved global cognition across diverse clinical populations.^69^

This study has some limitations. First, since the current data was drawn from a non-inferiority trial that compared two types of rTMS, there was no sham-TMS control group. To account for repeated assessments and practice effects, we calculated RCI_PE_ using published normative data, which was derived from adults without clinical diagnoses, and for the CVLT-II, only raw scores uncorrected for age were available.^50^ Given that age and clinical diagnosis affect the magnitude of practice effects,^70^ the cognitive benefits of rTMS treatment may be underestimated in older adults with depression because the practice effects may be different compared to the normative sample. Second, randomized participants who did not complete treatment or follow-up cognitive assessments had lower baseline cognitive performance. This limits generalizability of our results and could have biased our findings. Moreover, the high education level and predominantly white demographic of our participants may limit the generalizability of our findings to other older adult populations.

In conclusion, our study demonstrated limited cognitive benefits after bilateral standard rTMS or bilateral TBS for the treatment of LLD, with only a small proportion of participants showing reliable improvement in verbal learning. Future research examining cognitive effects of rTMS should include a clinically similar comparator group undergoing repeat cognitive assessment and evaluate cognitive changes both at group and individual levels. Comprehensive assessments should evaluate changes in specific cognitive tests, and measures of various aspects of executive functions like response inhibition, working memory, and cognitive flexibility should be included. Additionally, research should focus on adjunctive strategies and/or refined neuroanatomical targets to optimize the cognitive benefits of rTMS to effectively address both depressive and cognitive symptoms in LLD.
